# Blue Light Modulates Murine Microglial Gene Expression in the Absence of Optogenetic Protein Expression

**DOI:** 10.1038/srep21172

**Published:** 2016-02-17

**Authors:** Kevin P. Cheng, Elizabeth A. Kiernan, Kevin W. Eliceiri, Justin C. Williams, Jyoti J. Watters

**Affiliations:** 1 Departments of Biomedical Engineering, University of Wisconsin-Madison, Madison, WI 53706; 2 Comparative Biosciences, University of Wisconsin-Madison, Madison, WI 53706.

## Abstract

Neural optogenetic applications over the past decade have steadily increased; however the effects of commonly used blue light paradigms on surrounding, non-optogenetic protein-expressing CNS cells are rarely considered, despite their simultaneous exposure. Here we report that blue light (450 nm) repetitively delivered in both long-duration boluses and rapid optogenetic bursts gene-specifically altered basal expression of inflammatory and neurotrophic genes in immortalized and primary murine wild type microglial cultures. In addition, blue light reduced pro-inflammatory gene expression in microglia activated with lipopolysaccharide. These results demonstrate previously unreported, off-target effects of blue light in cells not expressing optogenetic constructs. The unexpected gene modulatory effects of blue light on wild type CNS resident immune cells have novel and important implications for the neuro-optogenetic field. Further studies are needed to elucidate the molecular mechanisms and potential therapeutic utility of blue light modulation of the wild type CNS.

Microglia, otherwise known as brain macrophages, are central nervous system (CNS) resident immune cells of erythromyeloid origin^1^,^2^. A large number of microglial studies over the past two decades have revealed these cells to play an intricate role in nearly every aspect of neurological function, development, and disease^3^. Microglial inflammatory activities have been implicated in nearly every neurological injury and disease including psychiatric disorders such as schizophrenia and depression^4^,^5^. Following CNS insult, microglia display an activated morphology and produce pro-inflammatory cytokines such as interleukin 1 beta (IL-1β) and tumor necrosis factor alpha (TNFα), as well as other pro-inflammatory molecules including nitric oxide (produced by inducible nitric oxide synthase; iNOS) and prostaglandins (produced by cyclooxygenase-2; COX-2)^6^. In parallel, microglia also produce a number of anti-inflammatory and neuroprotective/trophic factors such as interleukin 10 (IL-10), transforming growth factor beta (TGF-β), vascular endothelial growth factor (VEGF), and insulin-like growth factor (IGF-1) which activate pro-survival pathways and limit the magnitude of the damaging inflammatory response^7^,^8^. Microglia also produce interleukin 6 (IL-6) which, while traditionally thought of as pro-inflammatory, can be both neurotrophic and anti-inflammatory in the CNS^9^,^10^. Although acute microglial activation is a necessary aspect of the tissue repair response to injury and foreign pathogens, when their inflammatory activities become dysregulated, a state of chronic activation and neuroinflammation can result, even after the initial insult has subsided^3^,^6^. Chronic microglial activation leads to exaggerated neuronal death and dysfunction of neural circuits which has made microglia of particular interest in neurodegenerative diseases such as Alzheimer’s and Parkinson’s diseases, and amyotrophic lateral sclerosis (ALS) wherein pro-inflammatory cytokines are thought to contribute to disease progression^3^. These observations have led to the therapeutic targeting of neuroinflammation to treat neurodegenerative disease, and underscore the need for new tools to modulate and study these processes.

Blue light has become a routinely applied tool used across multiple disciplines with the advent of fluorescent microscopy and fluorescence assisted assays. Additionally, recent advances in optogenetic tools over the past decade have led to a dramatic increase in the use of blue light in non-fixed, live *in vitro* and *in vivo* applications, especially in neuroscience^11^,^12^. However, relatively little is known about the potential side effects of these levels of blue light on non-neuronal CNS cell types, including microglia, that do not express optogenetic proteins^13^. To date, the majority of studies utilizing blue light in a non-imaging or non-optogenetic application have focused on its use as a germicidal agent or in targeted carcinoma ablation^14^,^15^. These studies typically use high intensity and long duration exposures to deliver a high energy dose of light. In parallel, literature reports and clinical trials have suggested that red and near-infrared (NIR) light may have many therapeutic effects in their own rights, across a range of conditions including wound healing, arthritis, myocardial infarction, and various neuropathologies^16^. However, whether blue light has similar effects is not yet known.

In the present study, the effect of low levels of blue light on the expression of microglial inflammatory, trophic, and anti-inflammatory genes was assessed via qRT-PCR. We found that blue light altered basal gene expression in both immortalized and primary microglia when delivered in short, repetitive, low level doses. Application of blue light in this pattern also significantly decreased pro-inflammatory gene expression in response to treatment with bacterial lipopolysaccharide (LPS). Importantly, blue light delivered in an exposure pattern typical of optogenetic applications similarly reduced LPS-stimulated inflammatory microglial gene expression, demonstrating the need to consider previously unreported, off-target effects of blue light when using optogenetic methods in long term CNS studies.

## Results

### Blue light gene-specifically alters basal microglial gene expression

**Figure 1:**
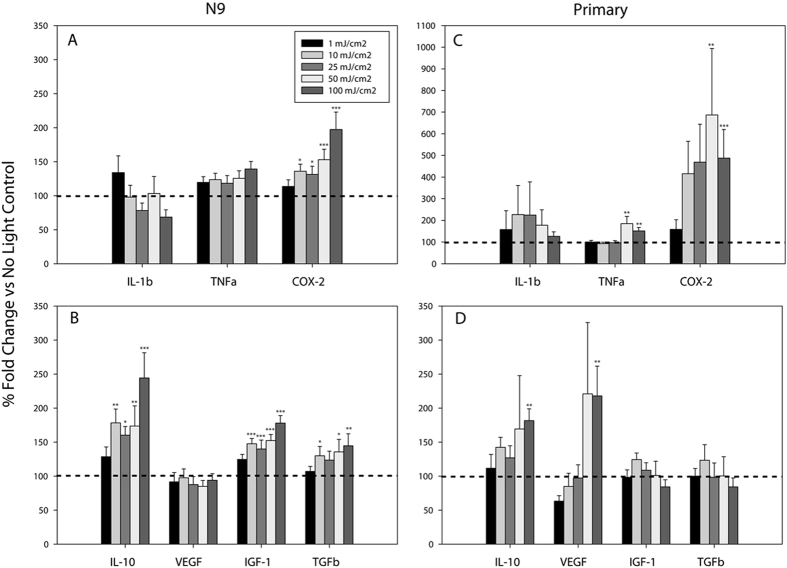
Blue light dose-dependently increases basal gene expression in naïve microglia. Blue light (450 nm) was delivered for 1 second per minute for 6 hrs at the indicated energy doses. Data are graphed as means + 1 SEM of light-induced % change in gene expression relative to that observed in the absence of light (dotted line). (**a**) Expression of pro-inflammatory and (**b**) anti-inflammatory/growth factor genes in N9 microglia (n = 6 each light condition). (**c**) Expression of pro-inflammatory and (**d**) anti-inflammatory/growth factor genes in primary microglia (n = 3 light doses 1, 10, 25, 50 mJ/cm^2^; n = 6 light dose 0, 100 mJ/cm^2^). *P < 0.05, **P < 0.01, ***P < 0.001 vs. no light control by Holm-Sidak test.

To carry out these studies, we first constructed a custom device consisting of 96 CREE XT-E royal blue LEDs configured into a standard 96-well plate array (see online methods). A custom MATLAB graphical user interface was created to allow for user-designed light delivery patterns of defined intensity, duration, and number of repetitions. To assess the effects of blue light on gene expression in naïve microglia, we exposed immortalized murine N9 and primary microglia to blue light doses between 0 and 100 mJ/cm^2^∙min by adjusting the LED intensity while keeping the duration of exposure constant at 1 second per minute over a 6 hour period. We found that blue light significantly altered basal N9 microglial inflammatory and growth/trophic factor gene expression levels in both a dose- and gene-specific manner (Fig. 1). mRNA levels for the pro-inflammatory cytokines *Il-1β* and *Tnfα* displayed no change in gene expression at any light intensity level although there was a dose-dependent increase in *Cox-2* gene expression starting at a dose of 10 mJ/cm^2^∙min (Fig. 1a). Expression of the anti-inflammatory and trophic factors *Il-10*, *Il-6, Igf-1*, and *Tgfβ* were all dose-dependently increased by blue light between 10 mJ/cm^2^∙min and 100 mJ/cm^2^∙min (Fig. 1b, *Il-6* Fig. S.1a). However, *Vegf* exhibited no change in expression at any light dose tested (Fig. 1b).

To further confirm these observations, we tested the effects of blue light in primary murine microglia. We found that although primary cells generally behaved similarly to N9 microglia, there were some notable gene-specific differences. For example, blue light induced statistically significant increases in *Cox-2* and *Tnfα* gene expression in primary microglia at doses of 50 and 100 mJ/cm^2^∙min (Fig. 1c), whereas only *Cox-2* gene expression was increased in N9 microglia. *Il-1β* expression was not altered in either primary (Fig. 1c) or N9 microglia (Fig. 1a). Whereas *Il-10* expression was increased by light in both N9 and primary microglia, it only reached statistical significance in primary cells at the maximum light dose delivered (100 mJ/cm^2^∙min, Fig. 1d). As in N9 microglia, while *Il-6* behaved similarly to *Il-10,* it did not achieve statistical significance (p = 0.181; Fig. S.1b). *Igf-1* and *Tgfβ* mRNA levels were unchanged in primary microglia at any light dose (Fig. 1d), although there were small but statistically significant increases in the expression of these genes in N9 microglia (Fig. 1b). Lastly, *Vegf* levels increased in primary microglia at the highest light dose tested (Fig. 1d), an effect that was not observed in N9 microglia (Fig. 1b).

### Blue light attenuates LPS-induced inflammatory gene expression

**Figure 2:**
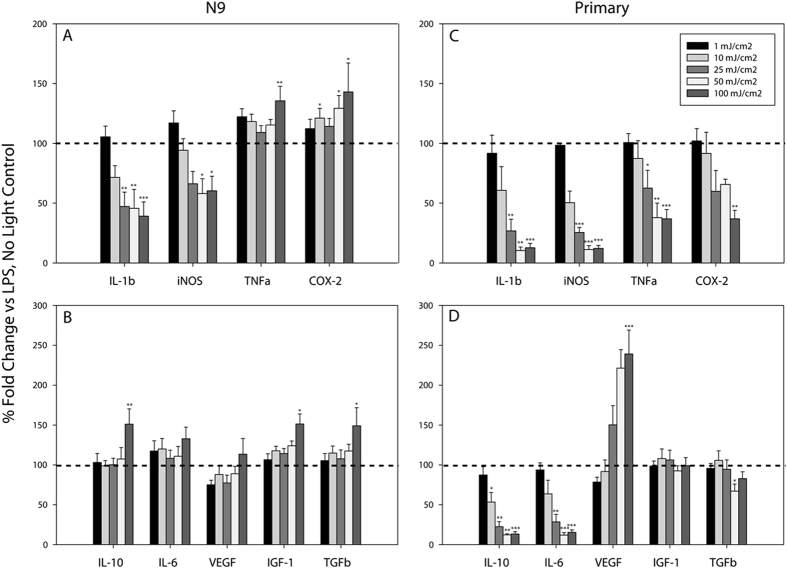
Blue light dose-dependently alters LPS-stimulated microglial inflammatory gene expression. Blue light (450 nm) was delivered for 1 second per minute for 6 hrs at the indicated energy doses, to cells treated with vehicle or 1 μg/mL LPS. Data are graphed as means + 1 SEM of light-induced % change in LPS-stimulated gene expression relative to that observed in the presence of LPS and absence of light (dotted line). (**a**) Expression of pro-inflammatory and (**b**) anti-inflammatory/growth factor genes in N9 microglia (n = 6 each light condition). (**c**) Expression of pro-inflammatory and (**d**) anti-inflammatory/growth factor genes in primary microglia (n = 3 light doses 1, 10, 25, 50 mJ/cm^2^; n = 6 light dose 0, 100 mJ/cm^2^). *P < 0.05, **P < 0.01, ***P < 0.001 vs. no light control by Holm-Sidak test or Tukey test (LPS-treated: Tgfβ, Igf-1, Cox-2).

Having directly observed alterations in basal microglial gene expression upon blue light exposure, we next wished to investigate the effects of light on gene expression in activated microglial cultures. To do this, we assessed gene responses following microglial exposure to LPS (1 μg/mL) in the presence and absence of light. The data are presented as magnitude of gene expression in the presence of LPS and light, graphed relative to an LPS exposed, no light control (dotted line). In N9 cells, light dose-dependently decreased LPS-induced expression of the pro-inflammatory genes *Il-1b* and *iNos* (Fig. 2a). Conversely, LPS-stimulated *Tnfα* and *Cox-2* expression exhibited dose-dependent increases in expression (Fig. 2a), similar to observations in naïve microglia (Fig. 1a). Additionally, LPS-induced *Il-10*, *Igf-1*, and *Tgfβ* expression was significantly augmented by exposure to blue light, while *Il-6* exhibited a non-significant trend to increase (p = 0.282; Fig. 2b). Similar to naïve N9 microglia (Fig. 1b), blue light did not alter LPS-stimulated *Vegf* mRNA levels (Fig. 2b).

Interestingly, the modulatory effects of blue light appeared to be more pronounced in primary microglia than in N9 microglia. In primary cultures, blue light significantly and dose-dependently decreased LPS-induced mRNA levels for *IL-1β*, *iNos, Tnfα*, and *Cox-2* (Fig. 2c). However, unlike the effects of light on *Il-10* and *Il-6* gene expression in naïve primary cultures (Fig. 1d) and LPS exposed N9 cells (Fig. 2b), blue light significantly reduced the expression of these genes in LPS-stimulated primary cultures (Fig. 2d). In agreement with the effects of blue light in naïve primary cells (Fig. 1d), blue light also augmented LPS-induced *Vegf* expression but had no effect on LPS-stimulated *Igf-1* or *Tgfβ* mRNA levels (Fig. 2d).

### Gene expression changes are reflected at the protein level

**Figure 3:**
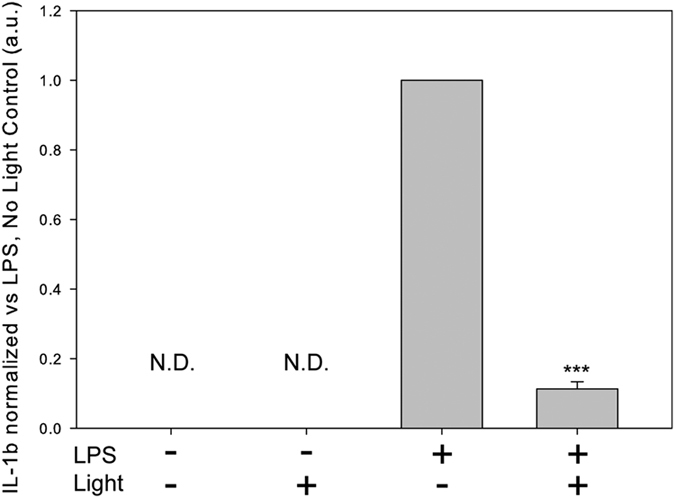
Blue light exposure reduces IL-1β cytokine levels in the culture medium of primary microglia treated with LPS. Primary microglia were exposed to blue light (450 nm) for 1 second per min at an intensity of 100 mW/cm^2^ and treated with either vehicle or LPS (1 μg/mL) for 6 hours. Data are graphed as means + 1 SEM of arbitrary units normalized to the LPS only condition. IL-1β levels were below the detection threshold of the assay and are designated as not detectable (N.D.). n = 3, ***P < 0.001 student’s t-test.

To determine whether blue-light induced anti-inflammatory effects on gene expression were also evident at the level of the protein, we measured the concentration of secreted IL-1β from primary microglia in the culture media supernatant following 6 hours of LPS (1 μg/mL) and blue light (100 mJ/cm^2^∙min). As expected, IL-1β was not detectable in either the control or light-only conditions, but there was a significant decrease in LPS-stimulated IL-1β levels in the supernatants from light-treated cultures (Fig. 3). Indeed, the relative magnitude of the decrease in IL-1β protein was consistent with that of the mRNA (~86% reduction IL-1β mRNA (Fig. 2C) vs. ~89% reduction IL-1β protein (Fig. 3)).

### Blue light does not affect microglial cell survival or promote DNA strand breaks

**Figure 4:**
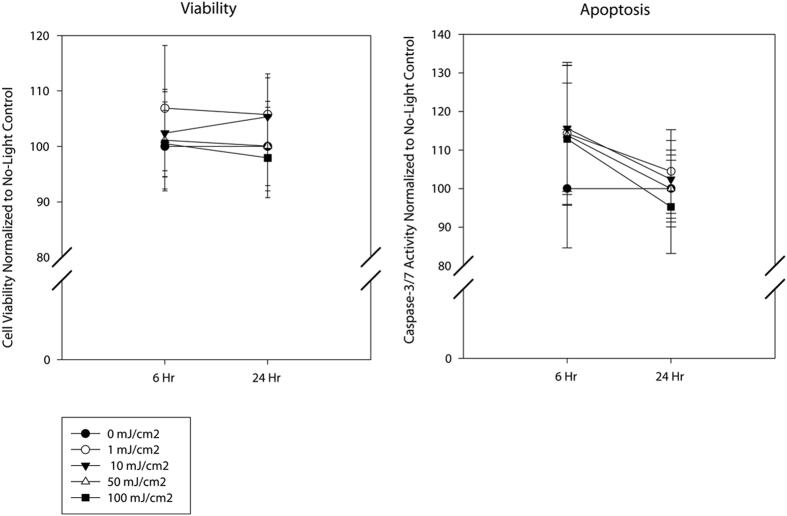
Blue light does not decrease microglial cell viability or promote apoptosis. N9 microglia were exposed to blue light (450 nm) for 1 second per minute for 6 hrs at the indicated energy doses and assayed immediately following or 18 hours post-exposure. Viability (left panel) and apoptosis (right panel) are graphed as means + 1 SEM assessed relative to the no light controls (0 mJ/cm^2^). No statistically significant differences in viability or apoptosis were detected.

Blue light can have potentially phototoxic and DNA damaging properties due to its higher energy content than longer wavelengths of light such as green or red. However, little is known about the effects of blue light when delivered at relatively low doses as was done here. To determine whether the reductions we observed in inflammatory gene expression were the result of promoting microglial cell damage, we performed two toxicity assays using the same light pattern and intensities. We evaluated cell viability and apoptosis 6 hr or 24 hrs following initiation of light exposure using a dual assay that measures both cell viability (using GF-AFC fluorescent dye activation) and apoptosis (using caspase-3/7 activity). We detected no significant changes in either viability (Fig. 4, left) or apoptosis (Fig. 4, right) in the light exposed samples as compared to the non-light exposed controls, even at the maximum light dose delivered (100 mJ/cm^2^∙min). It should be noted that while it was not statistically significant (p = 0.575, student’s t-test, n = 3), at the 6 hour time point there was an apparent ~16% increase in caspase-3/7 activity which was not evident after 24 hours. This could reflect the role of caspase-3 activity in regulating microglial activation^17^.

**Figure 5:**
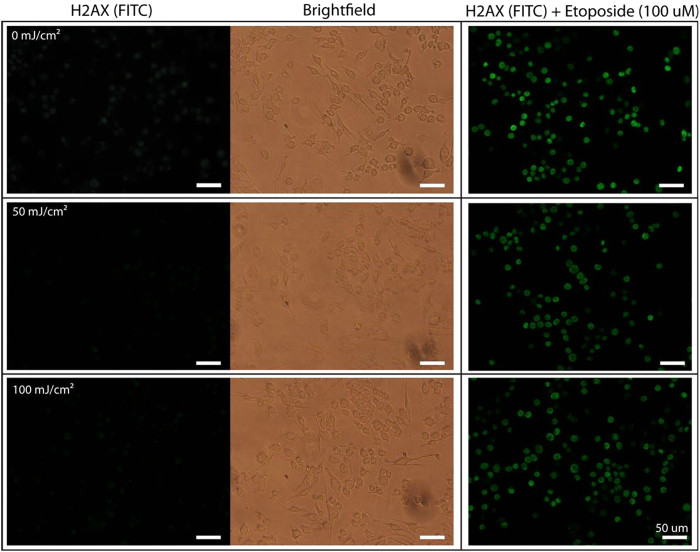
Blue light does not promote DNA damage in microglia. N9 microglia were exposed to no light (0 mJ/cm^2^; control, top row) or blue light (450 nm) at 50 mJ/cm^2^ (middle row) and 100 mJ/cm^2^ (bottom row) for 1 sec/min for 6 hours, after which time they were stained with anti-γ-H2AX antibodies (left column). Brightfield images of the same field-of-view are shown in the middle column. As a positive control for DNA strand breaks, cells were also exposed to 100 μM etoposide and stained with anti-γ-H2AX antibodies (right column). No dose of light tested induced DNA damage, and blue light did not further increase etoposide-induced DNA damage. Scale bar = 50 μm.

To evaluate potential changes in DNA damage due to blue light exposure, we used fluorescence microscopy to evaluate the levels of phosphorylated γ-H2AX immunoreactivity, an H2A histone variant that localizes to DNA strand breaks and becomes rapidly phosphorylated. N9 microglia were exposed 0, 50 or 100 mJ/cm^2^∙min via 1 sec/min exposures for 6 hrs, and assayed immediately thereafter. There were no detectable increases in phosphorylated γ-H2AX fluorescence that would be indicative of DNA damage in microglia exposed to either 50 mJ/cm^2^∙min or 100 mJ/cm^2^∙min as compared to the non-light exposed (0 mJ/cm^2^∙min) controls (Fig. 5, left and center panels). In addition, blue light did not appear to enhance the effects of 100 μM etoposide, a topisomerase II inhibitor that causes DNA strand breaks, administered as a positive control (Fig. 5, right panels).

### Optogenetic patterns of blue light also affect gene expression

We next wished to determine if optogenetic light delivery patterns of blue light had similar effects on microglial gene expression, as this information is critical for future therapeutic applications of optogenetics. Previously, we delivered blue light in 1 second boluses once per minute, but optogenetic approaches typically use rapid, short duration pulses at the time scales of neural electrical activity^18^,^19^. Thus, blue light at a dose of ~25 mW/cm^2^ was delivered in brief 15 ms pulses at a rate of 10 Hz to primary microglial cultures in the presence and absence of 1 μg/mL LPS. The number of pulses administered was calculated such that the total amount of energy delivered per minute was 100 mJ/cm^2^∙min, coinciding with the maximum light dose delivered in our previous studies.

**Figure 6:**
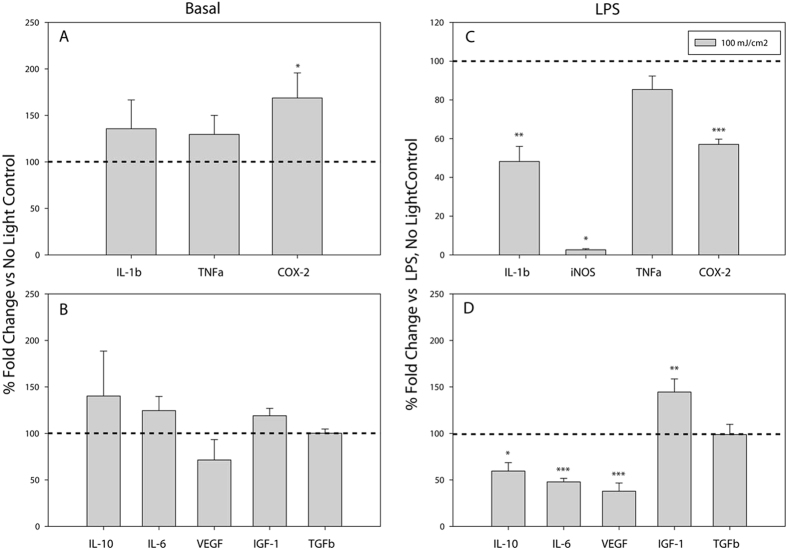
Blue light delivered in an optogenetic pattern decreases LPS-stimulated microglial inflammatory gene expression. The effect of optogenetic light delivery on the basal expression of (**a**) pro-inflammatory and (**b**) anti-inflammatory/growth factor genes (n = 6), or LPS-induced expression of (**c**) pro-inflammatory and (**d**) anti-inflammatory/growth factor genes (n = 6) is shown. 15 ms pulses of blue light (450 nm) was delivered at 10 Hz, (~25 mW/cm^2^) for 6 hrs to primary microglia treated with vehicle (**a**,**b**) or 1 μg/mL LPS (**c**,**d**). Data are graphed as means + 1 SEM of light-induced % change in gene expression relative to the no light controls (dotted line; **a**,**b**) or light-induced % change in LPS-stimulated gene expression relative to LPS alone in the absence of light (dotted line; **c**,**d**). *P < 0.05, **P < 0.01, ***P < 0.001 vs. respective to no light controls by Holm-Sidak test.

Whereas blue light delivered in an optogenetic pattern did not increase the basal expression (Fig. 6a,b) of most genes evaluated (i.e. *Il-10 p* = *0.458*, *Il-6 p* = *0.359*, *Igf-1 p* = *0.059*, *IL-1β p* = *0.277*, and *Tnfα p* = *0.171*), that of *Cox-2* reached statistical significance (Fig. 6a). *Vegf* mRNA levels were not statistically altered by optogenetic pattern light delivery in naïve microglia (Fig. 6b), despite their significant up-regulation with the previous pattern of light delivery (1 second boluses every minute) (Fig. 1d). In contrast, in LPS-activated microglia (Fig. 6c,d), the optogenetic light delivery pattern had effects on microglial gene expression similar to those observed using the non-optogenetic pattern. The LPS-induced expression of *Il-1β*, *iNos* and *Cox-2* were significantly reduced (Fig. 6c). The slight decrease in *Tnfα* mRNA levels did not reach statistical significance (p = 0.109; Fig. 6c). As was observed with the non-optogenetic light delivery pattern (Fig. 2d), LPS-stimulated *Il-10* and *Il-6* mRNA levels significantly decreased with optogenetic light delivery (Fig. 6d). Whereas LPS-stimulated *Vegf* expression also significantly decreased with optogenetic light delivery, LPS-induced *Igf-1* mRNA levels increased and *Tgfβ* levels were unchanged (Fig. 6d). These results are generally consistent with the gene expression effects of non-optogenetic light delivery (Fig. 2d).

## Discussion

In the present study we demonstrate that blue light gene-specifically alters the basal expression of inflammatory and neurotrophic genes in both immortalized and primary murine microglia. Further, we found that blue light delivered at these doses strongly reduces inflammatory gene expression in LPS-activated microglia in the absence of significant changes in cell viability, apoptosis, or DNA damage. Lastly, when blue light is delivered in a pattern typical of those commonly used in optogenetic studies, we observe similar decreases in inflammatory gene expression in LPS-activated microglia, suggesting that optogenetic light delivery causes previously unreported, off-target effects in cells not expressing the optogenetic construct. These observations have significant implications for the therapeutic use of optogenetics in the CNS.

The concept of using light to alter cellular behavior and function in the absence of an artificially introduced photo-activating molecule was first described in 1967 by Endre Mester, and is the basis of the field of photobiomodulation, also known as low level laser therapy (LLLT)^16^. LLLT today is performed almost exclusively within the 600–1000 nm wavelength range as these wavelengths have greater tissue penetration depths and are considered safer than shorter wavelengths since they carry less energy per photon^16^. Studies of the effect of red and NIR have been previously performed on monocytic cell types *in vitro* including macrophages and microglia. In RAW 264.7 macrophages, 780 nm light at doses of 2.2 J/cm^2^ decreased *Il-1β* and *Il-6* gene expression but had no effect on *Tnfα* or *iNos* in response to LPS^20^. Conversely, 632.8 nm light in a single bolus dose of 20 J/cm^2^ significantly reduced iNOS and TNFα protein levels in LPS-activated microglia^21^. However, the present study is the first to our knowledge to report reductions in the expression of inflammatory gene expression by blue light (450 nm) in mammalian cells. We found that short repetitive exposures to blue light at doses as low as 25 mJ/cm^2^∙min were able to significantly reduce the expression of *Il-1β*, *iNos*, *Tnfα*, and *Cox-2* regardless of whether it was delivered in a bolus or optogenetic pattern to LPS-stimulated primary microglia. However, it is important to note that optogenetic patterned blue light also gene-specifically increased the expression of some pro-inflammatory genes (*Cox-2*) in naïve microglia and decreased the expression of some anti-inflammatory/neurotrophic cytokines in activated microglia, suggesting biological complexity of blue light with regard to the overall activities of microglia.

The inflammatory genes assessed here play significant roles in promoting neuronal death and degeneration in a number of inflammatory, degenerative and traumatic injuries; thus, attenuated expression may have significant therapeutic relevance for a host of CNS disorders^3^,^22^. Specifically, elevated levels of the pro-inflammatory cytokine IL-1β have been observed in Alzheimer’s disease lesions in human post-mortem tissue, and IL-1β knockout mice exhibit resistance to LPS-induced Parkinson’s-like symptoms^3^,^23^. Exposure of microglial cultures to amyloid β-peptides dose-dependently increases the production of both TNFα and IL-1β, and elevated levels of TNFα have been detected in the cerebrospinal fluid of ALS patients^3^, suggesting that reducing these cytokines might be beneficial. Further, *Cox-2* and *iNos* knockout mice demonstrate reduced infarct volumes and neurologic deficits following ischemic injury^24^,^25^, indicating that these enzymes are also involved in neuronal injury in stroke. Conversely, while these inflammatory and potentially neurotoxic molecules are typically associated with pathologies, many of these molecules are also necessary for various aspects of healthy neuronal development and function. For example, both TNFα and COX-2 have critical roles in neuronal plasticity and learning and memory^26^,^27^. Further, creating potential imbalances in the time course and magnitude of pro-and anti-inflammatory gene expression may deregulate the CNS immune system, and contribute to pathology. Therefore, it is unclear at present, whether the blue light-induced increases in the basal expression of *Tnfα* and *Cox-2,* and/or decreases in anti-inflammatory gene expression in activated microglia observed here are ultimately beneficial or pathologic. However, these results are the first to suggest that such studies are indeed warranted.

In light of the strong links between pro-inflammatory molecules and neurodegenerative disease, and the significant reductions in inflammatory gene expression by blue light in activated microglia *in vitro*, the ability to use blue light to modulate microglial activities in a local region of the CNS, is of particular interest. It is possible that tools already in development for optogenetic research could be applied towards a novel, therapeutic use^28^,^29^. For example, delivering blue light from the electrodes used in deep brain stimulation methods already in use clinically to treat Parkinson’s disease patients may help extend the functionality of the probes by minimizing the microglial and inflammatory tissue responses around the probes, thereby extending the time of therapeutic utility of the probes. Indeed, the rapid miniaturization and concomitant reductions in cost of LEDs and electronics improves the potential for small implantable devices to be used to deliver potentially anti-inflammatory light paradigms to targeted volumes of neural tissue, thereby circumventing the problems associated with systemic drug delivery and CNS penetrance.

An important yet poorly understood aspect of microglial activities is their production and secretion of many trophic factors critical for normal brain development and homeostasis. Here, we evaluated microglial expression of *Vegf*, *Igf-1* and *Tgf-β*. IGF-1 is important for myelination and normal cognitive function^8^; microglial-derived IGF-1 in particular has been identified as a necessary molecule for survival of layer V neurons during postnatal development^30^. TGF-β is a potent suppressor of microglial activation and contributes to their maintenance in the quiescent/surveillant state in the healthy brain^31^. Thus, upregulated basal expression of *Igf-1* and *Tgfβ* by blue light suggests that light may not only exert beneficial effects by reducing microglial expression of inflammatory genes, but also by potentially increasing their production of growth factors beneficial to neural cells. Interestingly, the effects of blue light on neurotrophic factor gene induction differed between primary and N9 microglia; *Vegf* upregulation, but not *Igf-1* or *Tgfβ* was observed in primary cultures. VEGF is important for neuroplasticity, blood vessel formation, and neurogenesis in the developing brain, and it is a key factor in axon regeneration following traumatic injury^32^,^33^. Thus, it is tempting to speculate that blue light exposure may be a novel therapeutic approach to reduce inflammation and facilitate neuroregeneration following traumatic injury.

Primary and immortalized microglial cultures had gene-specific differences in their response to blue light. Although the underlying reasons for this are not yet known, it may result from the retroviral-mediated expression of the oncogene used to immortalize this cell line^34^. Alternatively, we have previously reported on differential microglial responses between microglia derived from differing brain regions, as well as different mouse strains^35^,^36^. Here, the primary cultures were prepared from the whole brains of postnatal day ~4–7 C57Bl/6 mice, an inbred strain, whereas N9 microglia are derived from the cerebral cortex of embryonic day ~12–13 of the outbred ICR/CD1 strain. Since we have previously shown that basal microglial gene expression differs greatly over early postnatal development^37^, there may be similar differences in microglial responses between the last gestational week and the first postnatal week as well.

The molecular mechanism(s) whereby blue light exerts effects on microglial gene expression remains unclear, although there are some lines of evidence to suggest that reactive oxygen species (ROS) may play a role. Red/NIR photobiomodulation stimulates mitochondrial cytochrome c oxidase which accelerates the electron transport chain^16^. Mitochondrial ROS, natural byproducts of the electron transport chain, are the primary effectors of red/NIR light stimulation in certain cell types^38^. Similarly, blue light can also induce ROS generation through the photoreduction of flavins, and studies using confocal laser scanning microscopy have demonstrated production of H_2_O_2_ from cellular flavin-containing oxidases^39^,^40^. Photoreduction of flavins readily leads to the production of H_2_O_2_ via cellular reducing agents^41^. Traditionally, ROS have carried a negative connotation in terms of cellular health, as they are often associated with carcinogenesis, DNA damage, cell cycle arrest, and induction of apoptosis^42^. Indeed, H_2_O_2_ itself has been used in some studies as an activating agent in inflammatory cell types, and as an inducer of apoptosis^43^,^44^. However, over the last decade, a growing body of evidence demonstrates H_2_O_2_ to be an important second messenger in many cellular functions^45^. Contrary to its use as a cell-killing agent, low doses of H_2_O_2_ elicit protective effects via activation of anti-apoptotic and pro-survival pathways^42^,^46^. In addition, as a key molecule in determining the intracellular redox state, H_2_O_2_ can exert a host of effects through direct and indirect inhibition of redox sensitive transcription factors such as NF-κB, HIF-1α, and AP-1^47^. Thus, the beneficial effects of blue light stimulation may involve the production of H_2_O_2_ that inhibits the activity of key pro-inflammatory transcription factors.

Currently, it is not clear whether the effects reported here *in vitro* also occur *in vivo.* Indeed, common components of cell culture media formulations may include molecules such as riboflavin that can absorb blue light and lead to the extracellular production of ROS. Important in this regard however, is that flavin-based molecules are found within all mammalian cells and include critical enzymatic co-factors such as flavin adenine dinucleotide (FAD) and flavin mononucleotide (FMN). Thus, while the current work describes the effects of blue light on microglia *in vitro*, it is likely that ubiquitous endogenous flavins *in vivo* will have similar responses to blue light. Given the increasing application of blue light in long-term scientific studies our work raises the important consideration that while only specific cell types may express the exogenously delivered light-sensitive protein of interest, all cells likely have an endogenous, blue light-responsive mechanism that has the capacity to alter their gene expression/function. To better understand the effects that blue light may have on other CNS cell types as well as on non-neuronal tissues, further investigation using large-scale gene and protein analyses coupled with functional assays *in vivo* are required.

## Methods

### Cell Culture

Murine N9 microglial cells were a kind gift from Dr. Paula Ricciardi-Castagnoli (University of Milan, Italy). They were routinely cultured in Dulbecco’s modified Eagle’s medium (DMEM; Hyclone, Logan, UT) supplemented with 10% fetal bovine serum (FBS; Hyclone) in 10 cm tissue culture treated dishes (BD-Falcon, San Jose, CA). Cells were passaged upon reaching 80–90% confluency, and were used prior to passage 6.

#### Primary cultures

C57Bl/6 mice were purchased from Jackson Labs (Bar Harbor, Maine). All animals were maintained in an AALAC-accredited animal facility with a 12-hr light/dark cycle regime with access to food and water ad libitum. All experiments were approved by the University of Wisconsin Madison Institutional Animal Care and Use Committee. Pups were sacrificed between postnatal days ~3–7 and mixed primary cultures were prepared as we have previously described^46^. Microglia were harvested by shaking and cultured in DMEM supplemented with 10% FBS and 100 U/mL penicillin-streptomycin (Corning-CellGro, Manassas, VA) for one night prior to experimentation.

### Blue light treatment of cells in 96-well plates

We developed a light emitting diode (LED) device containing 16 banks of programmable LEDs. Each bank holds 6 individual LEDs for a total of 96; each aligned with the center of an individual well of a standard 96-well tissue culture plate. To apply blue light we used CREE XT-E Royal Blue LEDs with a peak emission at 450 nm (20 nm FWHM). The LEDs are powered by four Texas Instruments LM3407 drivers with pulse width modulated dimming current and a maximum driving current of 350 mA. The driver current is controlled using an on-board Arduino ATmega32U4 (Arduino) microcontroller containing a mini-USB programmable input. Output intensity was measured using an X-Cite XR2100 optical power meter equipped with an XP750 sensor (Lumen Dynamics). A MATLAB GUI was developed alongside the LED array that allows the user to individually define intensity, duration, and the number of repetitions for each bank of LEDs. A custom plastic mounting was created using a 3D printer (MakerBot) that isolated the light produced by each individual LED to a single well of a 96-well plate. Further, all experiments were performed in black-walled, tissue culture treated 96-well plates (BD-Falcon) to prevent light contamination across wells.

For experimentation, N9 microglia were plated overnight in at a density of 10,000 cells/well. Primary microglia were seeded at a density of 40,000 cells/well. The following day the cells were treated with either vehicle (Hank’s balanced salt solution (HBSS); Corning-CellGro) or LPS (1 μg/mL; Sigma-Aldrich, St. Louis, MO) and exposed immediately thereafter to either no light (control) or blue light, with an exposure paradigm of 1 sec/min for a total of 6 hours at the light doses indicated in the figures. At the end of the 6 hour period the cells were harvested in TRI-Reagent (Sigma-Aldrich) for gene expression analyses.

### RNA extraction/reverse transcription

Total RNA was extracted according to the manufacturer’s instructions using TRI-Reagent. 1 μL Glycoblue (Ambion, Grand Island, NY) was added during the isopropanol precipitation to assist with visualization of the pellet. cDNA synthesis reactions were performed with MMLV Reverse-Transcriptase (Life Technologies, Grand Island, NY), using between 200 ng and 1 μg of total RNA starting material. The cDNA was then diluted in nuclease-free ddH_2_O to a final concentration of 5 ng/uL of initial RNA concentration to maintain consistency among samples.

### Quantitative PCR

Real-time PCR was performed using Power SYBR Green (Life Technologies) on either an ABI StepOne or ABI 7500 Fast system (Applied Biosystems, Grand Island, NY) as we have described before. Primers (Table 1) were designed to span introns whenever possible and NCBI BLAST was used to confirm their gene specificity. Further verification of each PCR reaction was performed by observation of a single peak dissociation curve whose Tm approximated that expected of the designated amplicon. Primer efficiency was evaluated by serial dilutions, and relative gene expression for all genes was determined using the standard curve method^47^. All samples were run in duplicate and C_T_ values were averaged and normalized against ribosomal 18S RNA to control for equal loading.

### ELISA measurement of IL-1b cytokine in culture supernatant

Primary microglia were plated overnight at a density of 40,000 cells/well in black-walled 96-well plates and exposed to 100 mJ/cm^2^∙min for 6 hours in the presence or absence of LPS (1 μg/mL). Immediately following light exposure cell culture supernatant was collected, spun at 1000 g for 5 minutes to remove cellular debris, and frozen at −80 °C for later processing. In order to quantify IL-1β levels in the supernatant a sandwich-based Mouse IL-1β/IL-1F2 DuoSet ELISA kit (RnD Biosystems) was used following the kit protocol and quantified using a Synergy HT (BioTek, Winooski, VT) plate-reader. Concentration of unknown samples was determined from a standard curve analysis using SigmaPlot 11.0 (Systat).

### Cell viability and apoptosis assessments

N9 microglia were plated overnight at a density of 10,000 cells/well in black-walled 96-well plates and were exposed to 0, 1, 10, 50, 100 mJ/cm^2^∙min of blue light for 6 hrs using the 96-LED device in the presence or absence of LPS (1 μg/mL). Cells were harvested either immediately (6 hrs) or 18 hrs after light exposure ceased (24 hrs) to determine if detrimental effects would manifest at some time after light exposure. Viability and apoptosis were assessed using the ApoLive-Glo (Promega, Madison, WI) multiplex assay, as per the manufacturer’s instructions. Viability was measured using glycyl-phenylalanyl-aminofluorocoumarin (GF-AFC) fluorescent dye activation. Apoptosis was measured in the same well by a caspase-3/7 activity-dependent luciferase reaction. Reagents for each respective reaction were incubated for 30 minutes. Fluorescence and luminescence signals were read on a Synergy HT plate reader. Control experiments to characterize the sensitivity and range of the assay on our plate reader were performed using serial dilutions of cells treated with staurosporine (20 nM to 10 μM) to induce apoptosis and ionomycin (200 nM to 100 μM) to reduce cell viability, as per the manufacturer’s recommendation.

### H2AX DNA damage stain

DNA damage was assessed using the OxiSelect DNA Double Strand Break (DSB) staining kit (Cell BioLabs, San Diego, CA). N9 microglia were plated in black-walled 96-well plates at a density of 10,000 cells/well. The following day the cells were exposed to 0, 50, or 100 mJ/cm^2^∙min for 6 hours using the 96-LED device with half of the wells for each light dosage receiving etoposide (100 uM; Cell BioLabs) during the last 1 hour to serve as positive controls. Cells were then immediately fixed and stained as per kit instructions. Fluorescent staining was visualized on a Nikon TS-100 microscope equipped with a B-2E/C filter cube (Nikon, Melville, NY) with illumination provided by a 120 W light source (XCite 120 lamp; Lumen Dynamics, Mississauga, ON, Canada). Images were captured using a Nikon DS-L1 camera system; illumination intensity and exposure time were kept constant across all images.

### Optogenetic light pattern delivery in 12-well plates

To deliver patterns of light typical of optogenetic experiments, a Sutter Lambda DG-4 equipped with a Semrock 465/15 nm bandpass filter was used to deliver blue light through the optical path of a Nikon TE-Eclipse microscope (Nikon). Light was passed through an open slot of the objective turret which matched the diameter of a single well of a 12-well plate (Thermo-Nunc, Waltham, MA). Custom black acrylic dividers were created to isolate each well and prevent light contamination across wells. The culture environment was maintained on the microscope stage at 37 °C with 5% CO_2_ by a Live-Cell incubator (Pathology Devices, Westminster, MD). The DG-4 shutter was controlled through the open sourced microscope control software MicroManager (https://www.micro-manager.org/) using a custom Beanshell script.

Cell culture was performed as described previously. Primary microglia were seeded at a density of 300,000 cells/well in a standard 12-well plate. The following day the cells were placed on the microscope set-up and exposed to light either in the presence or absence of LPS (1 μg/mL). The optogenetic light pattern consisted of 15 ms pulses delivered at a rate of 10 Hz for 6 hours. The intensity measured at the cells was ~25 mW/cm^2^. The number of pulses was calculated such that the total amount of energy delivered per minute was 100 mJ/cm^2^ to match that of the other bolus light delivery protocol. Immediately following exposure, the cells were placed in TRI reagent for further processing for gene expression analysis.

### Statistical Analysis

Statistical analyses were carried out using SigmaPlot 11.0 (Systat, San Jose, CA) by One-Way Repeated Measures ANOVA followed by a Holm-Sidak post-hoc test unless otherwise specified. The gene expression data (means + 1 SEM) are graphed relative either to no light exposure controls, or no light exposure LPS-treated controls. Statistical significance was set at P < 0.05. Data represent the averages of at least 3–6 independent experiments as indicated in the figure legends, and each treatment was performed in duplicate within each individual experiment. *P < 0.05, **P < 0.01 and ***P < 0.001 versus the respective control as indicated in each figure.

## Additional Information

**How to cite this article**: Cheng, K. P. *et al.* Blue Light Modulates Murine Microglial Gene Expression in the Absence of Optogenetic Protein Expression. *Sci. Rep.* **6**, 21172; doi: 10.1038/srep21172 (2016).

## Supplementary Material

Supplementary Figure 1

## Figures and Tables

**Table 1:** qPCR primer sequences.

Gene	Forward Primer	Reverse Primer
*18S*	CGGGTGCTCTTAGCTGAGTGTCCCG	CTCGGGCCTGCTTTGAACAC
*Il-1b*	TGTGCAAGTGTCTGAAGCAGC	TGGAAGCAGCCCTTCATCTT
*Tnf-α*	TGTAGCCCACGTCGTAGCAA	AGGTACAACCCATCGGCTGG
*Inos*	TGACGCTCGGAACTGTAGCAC	TGATGGCCGACCTGATGTT
*Cox-2*	CAGGTCATTGGTGGAGAGGTGTAT	CCAGGCACCAGACCAAAGACTT
*Il-10*	GCCTTATCGGAAATGATC CA	TCTCACCCAGGGAATTCCAAA
*Il-6*	CCACTTCACAAGTCGGAGGC	GCCATTGCACAACTCTTTTCTCA
*Vegf*	TTGAGACCCTGGTGGACATCT	CACACAGGACGGCTTGAAGA
*Tgf-β*	GGTTCATGTCATGGATGGTGC	TGACGTCACTGGAGTTGTACGG
*Igf-1*	CAAGACTCAGAAGTCCCCGT	ACTTCCTTTCCTTCTCCTTTGC
