# Supplementary material for: Blue Light Modulates Murine Microglial Gene Expression in the Absence of Optogenetic Protein Expression
Source: Sci Rep. 2016 Feb 17;6:21172. doi: 10.1038/srep21172 (PMC4756664; doi:10.1038/srep21172)
Supplement: Supplementary Figure 1 [file srep21172-s1.pdf]

Blue Light Modulates Murine Microglial Gene Expression in the Absence of Optogenetic Protein Expression

Supplementary Info

Kevin P. Cheng[1], Elizabeth A. Kiernan[2], Kevin W. Eliceiri[1], Justin C. Williams[1] and *Jyoti J. Watters[2]

Departments of Biomedical Engineering[1] and Comparative Biosciences[2], University of Wisconsin-Madison, Madison, WI 53706

Corresponding Author: Jyoti J. Watters, Department of Comparative Biosciences, 2015 Linden Drive, Madison, WI 53706. Tel: 608-262-1016, Fax: 608-263-3926, Email: jjwatters@wisc.edu

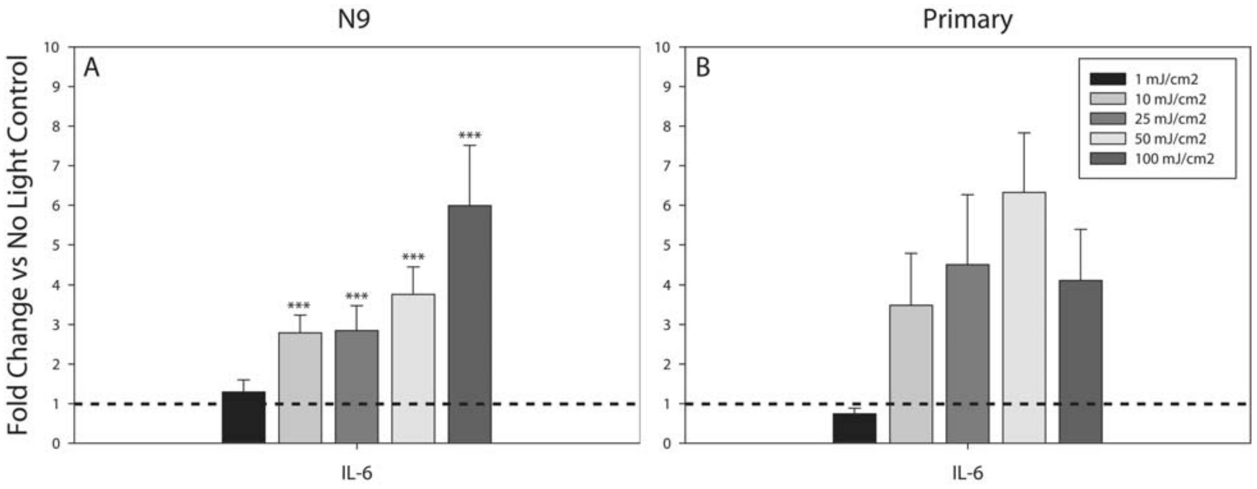

Supplementary Figure 1: Effect of blue light on basal Il-6 gene expression. *Blue light (450 nm) was delivered for 1 second per minute for 6 hrs at the indicated energy doses. The data were analyzed using the ΔΔCt method (Ct values fell outside of the linear range of the standard curve), and are graphed as means* $\pm$ *1 SEM of the light-induced % change in gene expression relative to that observed in the absence of light (dotted line). Il-6 gene expression in* ***a****) N9 microglia (n=6 each light condition) and* ***b****) primary microglia (n=3 light doses 1, 10, 25, 50 mJ/cm*^{2}*; n=6 light dose 0, 100 mJ/cm*^{2}*). **P < 0.01, ***P < 0.001 vs. no light control by Holm-Sidak test.*
